# Optimized Ultrasound-Assisted Enzymatic Extraction of Phenolic Compounds from *Rosa canina* L. Pseudo-Fruits (Rosehip) and Their Biological Activity

**DOI:** 10.3390/antiox11061123

**Published:** 2022-06-06

**Authors:** Alexandru Nicolescu, Mihai Babotă, Leilei Zhang, Claudiu I. Bunea, Laura Gavrilaș, Dan C. Vodnar, Andrei Mocan, Gianina Crișan, Gabriele Rocchetti

**Affiliations:** 1 Department of Pharmaceutical Botany, “Iuliu Hațieganu” University of Medicine and Pharmacy, Gheorghe Marinescu Street 23, 400337 Cluj-Napoca, Romania; alexandru_s_nicolescu@yahoo.com (A.N.); mihai.babota@umfcluj.ro (M.B.); gcrisan@umfcluj.ro (G.C.); 2 Department for Sustainable Food Process, Università Cattolica del Sacro Cuore, 29122 Piacenza, Italy; leilei.zhang@unicatt.it; 3 Faculty of Horticulture, University of Agricultural Sciences and Veterinary Medicine Cluj-Napoca, Calea Mănăştur 3-5, 400372 Cluj-Napoca, Romania; claus_bunea@yahoo.com; 4 Department of Bromatology, Hygiene, Nutrition, “Iuliu Haţieganu” University of Medicine and Pharmacy, 6 Pasteur Street, 400349 Cluj-Napoca, Romania; laura.gavrilas@umfcluj.ro; 5 Institute of Life Sciences, Faculty of Food Science and Technology, University of Agricultural Sciences and Veterinary Medicine, 400372 Cluj-Napoca, Romania; dan.vodnar@usamvcluj.ro; 6 Laboratory of Chromatography, Institute of Advanced Horticulture Research of Transylvania, University of Agricultural Sciences and Veterinary Medicine, 400372 Cluj-Napoca, Romania; 7 Department of Animal Science, Food and Nutrition, Università Cattolica del Sacro Cuore, Via Emilia Parmense 84, 29122 Piacenza, Italy; gabriele.rocchetti@unicatt.it

**Keywords:** *Rosa canina*, UAE, EAE, antioxidants, DoE, phenolic profiling

## Abstract

Two techniques, namely, optimized ultrasound-assisted extraction (UAE) and enzyme-assisted extraction (EAE), were used to promote the extraction of phenolic compounds from the pseudo-fruits of *Rosa canina* L. (RC). For UAE, an optimization process based on the design of experiment (DoE) principles was used for determining the dependence between three variables (i.e., time of extraction, ultrasound amplitude, and the material-to-water ratio) and the total phenolic content of the samples. For EAE, a 2:1:1 pectinase, cellulase, and hemicellulase enzymatic blend was used as pre-treatment for optimized UAE, inducing a higher total phenolic content. The untargeted phenolic profiling approach revealed a great abundance of lower molecular weight phenolics (1.64 mg Eq./g) in UAE-RC extracts, whilst gallic acid (belonging to hydroxybenzoic acid derivatives) was the most abundant individual compound of both extracts. The unsupervised multivariate statistics clearly discriminated the impact of enzymatic pre-treatment on the phenolic profile of RC pseudo-fruits. Finally, Pearson’s correlation coefficients showed that anthocyanins, phenolic acids, and tyrosol derivatives were those compounds mostly correlated to the in vitro antioxidant potential of the extracts, whilst negative and significant (*p* < 0.05) correlation coefficients were recorded when considering the enzymatic inhibition activities. The highest enzyme-inhibitory activity has been identified against α-glucosidase, which indicates an antidiabetic effect.

## 1. Introduction

**Figure 1:**
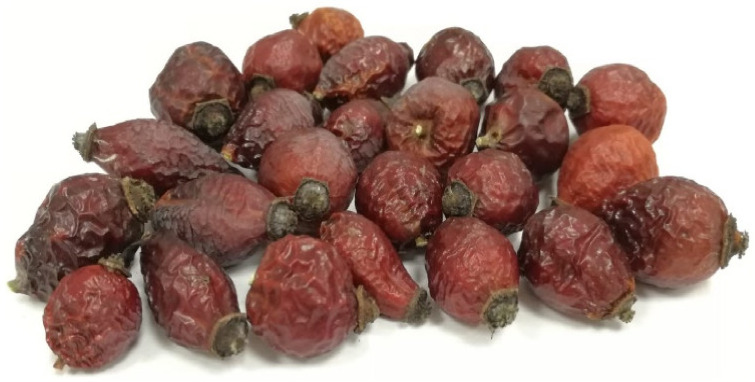
The appearance of dried *Rosa canina* L. pseudo-fruits used for extraction.

Considering that nowadays the use of herbal resources for nutraceutical purposes is intensively promoted based both on ethnopharmacological and scientific evidence, the research in this field is growing constantly [1]. In the Rosaceae family, the genus *Rosa* comprises more than 100 species, spread across Europe, Asia, and North America, their therapeutic and nutraceutical benefits being recognized and exploited for centuries [2]. Among them, *Rosa canina* L. (Figure 1) stands out as an important plant in European folk medicine, given its curative and prophylactic properties for infections, fever, and gastrointestinal and kidney disorders [3,4]. The fruits are known for possessing a high amount of vitamin C and polyphenolic compounds, being used as a herbal remedy. Additionally, the plant has an importance not only for its medical applications but also for being used in the cosmetic and food industry, including as part of several beverages [2]. Other *Rosa* species are recognized as medicinal; according to recommendations of Russian Pharmacopoeia, fruits of *R. acicularis*, *R. davurica*, *R. beggeriana*, *R. fedtschenkoana*, *R. rugosa*, and *R. majalis* are used as poly-vitamin sources, while the European Medicine Agency (through the Herbal Medicinal Products Committee) recommends the use of dried petals obtained from *R. centifolia*, *R. gallica*, and *R. damascena* as remedies for mild inflammation of the skin or lining of the mouth and throat [5,6].

Several studies highlighted the value of bioactive fractions obtained from the fruits of *R. canina* using different extractive techniques (i.e., maceration, infusion, decoction, percolation), ultrasound-assisted extraction (UAE) being recognized also as suitable for a good recovery of phenolic compounds from this matrix [4,7,8,9]. UAE is one of the most popular unconventional technologies currently used for extraction of polyphenols from a wide range of plant matrices due to its increased efficiency and safety. The main processes involved in UAE (i.e., cavitation, cell wall disruption, thermic effect) lead to short extraction times, use of small amounts of solvent/plant material, and increase the extraction yields, it being observed that the output of the extractive method may vary depending on the extraction parameters [10,11]. Moreover, UAE is considered a versatile extractive method, especially through the fact that its advantages can be augmented by coupling with other extractive techniques [12].

Thus, the present work was focused to develop an optimized extractive method for the phenolic fraction contained in *R. canina* fruits using UAE and enzyme-assisted extraction (EAE), aiming to establish and describe the influence of extraction parameters on the quality of the extracts obtained through these methods. Additionally, the extracts were further evaluated for their individual phenolic content and bioactive potential (in vitro antioxidant and enzyme-inhibitory activities) to study the correlation between extraction procedures and phenolic and bioactive profiles of these herbal preparations obtained from rosehip.

## 2. Materials and Methods

### 2.1. Plant Material

The plant material needed for the study (*Rosa canina* pseudo-fruits) was collected in October 2020 near the southern part of Cluj-Napoca (Cluj County, Cluj-Napoca, Romania) and then directly subjected to a controlled drying process according to an optimized process that was established by Moldovan et al., using hot air at a temperature of 60 °C for exactly 30.4 h [13]. After being dried at a constant mass, the plant material (Figure 1) was kept in the freezer at the Pharmaceutical Botany Department of “Iuliu Hațieganu” University of Medicine and Pharmacy of Cluj-Napoca until the extraction phase.

### 2.2. Extraction Procedure

To begin the extraction procedure, the obtained plant material was powdered using a laboratory mill (Grindomix^®^ GM 200, Retsch Gmbh., Haan, Germany), at 10,000 rpm for 5 min in total, and the uniform granulometry of the powder was assured by manually passing it through a 1 mm sieve (a standard according to PhEur 10.6). For this study, only water has been taken into consideration as extraction solvent, due to the fact that previous studies showed its importance as an environmentally friendly solvent with high efficiency on the recovery of antioxidant phytochemicals [14,15]. UAE was carried out using a SFX 150 Sonifier (Branson Ultrasonics Corporation, Brookfield, Connecticut, United States of America) equipped with a tapered microtip with a 3.2 mm diameter. The experimental design of the optimization process was accomplished considering three independent process variables, as following: the ultrasound amplitude (20, 30, and 40%), the exposure time (10, 30, and 50 min), and the material sample-to-liquid solvent ratio or SLR (1:10, 1:15, and 1:20). Considering every ratio, 1.5, 2, or 3 g of RC powder were exactly weighed and mixed with 28.5, 28, or 27 mL of distilled water, respectively, to ensure a total of 30 g of extraction mixture. During the UAE, the microtip was submersed at exactly 2 cm in the extract, in the same 50 mL capacity beaker; an ice bath was constantly used to avoid heating of the samples, and a magnetic stirrer was used for assuring the homogenizing of the samples. For every determination, the total power used by the ultrasounds (in watts) was also recorded, which was in accordance with the used amplitude.

Every obtained mixture was centrifuged; the supernatant was collected and subsequently filtered through cotton and paper filters to assure a clear solution. After establishing the optimal extraction parameters, a triplicate of optimized extracts (ORC) was obtained, and then they were freeze-dried and kept in a desiccator at room temperature until further analysis. Likewise, the same optimal parameters were used to obtain a triplicate of optimized extracts (ERC) but this time with a pre-treatment phase consisting of EAE. For this additional step, an enzymatic blend consisting of pectinase, cellulase, and hemicellulase was used, in a 2:1:1 ratio, having the following activities: 0.6 U/mL pectinase, 0.3 U/mL cellulase, and 0.3 U/mL hemicellulase. A constant 5.6 pH was assured using phosphate buffer, and EAE was practically realized by mixing the powder with the reaction mixture (enzyme blend in phosphate buffer) in a 50 mL Falcon tube at a constant temperature of 50 °C under constant shaking at 500 rpm using a Thermo-Shaker for 60 min. These parameters, along with the enzymes, were chosen considering previous studies that aimed to recover total phenolic compounds by means of EAE, with slight modifications [16,17,18]. After the end of the incubation time, the samples were subjected to UAE, using the same optimal parameters.

### 2.3. Design of Experiments

For the design of the experiments (DoE), the MODDE 13.0 software (Sartorius Stedim Data Analytics AB, Umeå, Sweden) was used [19]. This software allowed the determination of the effect of experimental variability and the optimal experimental parameters. For this study, a D-optimal type of DoE was chosen to benefit from the advantages of this family of designs. Being computer-generated using an automatic algorithm in MODDE, D-optimal designs are able to identify the best group of experiments in a candidate set, covering the largest possible volume of the experimental region for certain specifications of factors and responses, in contrast to classical response surface methodology approaches [20].

After finishing the experimental runs suggested by the software, the analysis of data was accomplished by evaluating raw data, regression analysis, and model interpretation. Finally, MODDE optimizer and determined response contour plots were used for the optimization step [20].

### 2.4. Total Phenolic Content (TPC)

Total phenolic content, or TPC, was determined based on the Folin-Ciocalteu method, adapted to a microplate reader, using a modified assay described by Babotă et al. [21]. Briefly, 20 µL of triplicate diluted samples were mixed with 100 µL of diluted Folin–Ciocalteu reagent (1:9, *v/v*) and shaken vigorously. After 3 min, 80 µL of 1% Na_2_CO_3_ solution was added, and after 30 min of incubation at room temperature the absorbance was read at 760 nm. The TPC was determined as a response parameter for the studied samples but also for the final freeze-dried extracts. The results were expressed as milligrams of gallic acid equivalents per gram of dried plant material or per gram of dried extract for ORC and ERC, as mg GAE/g.

### 2.5. UHPLC-HRMS Analysis of Phenolic Profile

The ORC and ERC lyophilized extracts (100 mg) were dissolved in 2 mL of water, centrifuged at 6000× *g* for 10 min at 4 °C, and then filtered through 0.22 μm cellulose syringe-filters. Thereafter, the filtered supernatants were transferred into UHPLC vials for instrumental analysis. The untargeted phenolic profiling was carried out by high-resolution mass spectrometry (HRMS) using a Q-Exactive™ Focus Hybrid Quadrupole-Orbitrap Mass Spectrometer (Thermo Scientific, Waltham, MA, USA) coupled to a Vanquish ultra-high-pressure liquid chromatograph (UHPLC) according to a heated electrospray ionization (HESI)-II probe (Thermo Scientific, Waltham, MA, USA). A gradient of water-acetonitrile (both LC-MS grade, from Sigma-Aldrich, Milan, Italy) from 6 up to 94% acetonitrile in 35 min was used for chromatographic separation, using 0.1% formic acid as phase modifier. The UHPLC was based on the utilization of a Waters BEH C18 column (2.1 × 100 mm, 1.7 μm). The mass spectrometry conditions were adapted from a previously published work [22]. Briefly, the flow rate was 200 μL/min; the injection volume was 6 μL; the full scan MS-data-dependent (Top *n* = 3) MS/MS mode was used for ion acquisition in the range 80–1200 *m/z*, with a positive ionization mode and a mass resolution of 70,000 FWHM. The automatic gain control target (AGC target) and the maximum injection time (IT) of the Orbitrap were 1 × 10^6^ and 200 ms, respectively. In the data-dependent MS/MS mode, the full scan mass resolution was reduced to 17,500 at *m/z* 200, with an AGC target value of 1 × 10^5^, maximum IT of 100 ms, and isolation window of 1.0 *m/z*, respectively. Three typical normalized collision energies were used for fragmentation, namely, 10, 20, and 40 eV. The HESI parameters are adapted from a previous work [23]. The raw data (.RAW files) were then processed using the software MS-DIAL (version 4.80) [24], and the annotation was performed via spectral matching against the comprehensive databases FooDB and Phenol-Explorer. For the identification step, a tolerance for mass accuracy of 5 ppm was used, and this was realized according to both isotopic pattern and spectral matching. Therefore, a level 2 of confidence in annotation (typical for untargeted metabolomics experiments) was achieved. Finally, regarding the semi-quantitative phenolic contents, the cumulative intensity values of the different phenolic classes were converted into semi-quantitative data, exploiting hydroalcoholic standard solutions of pure compounds (Extrasynthese, Lyon, France) analyzed under the same instrumental conditions. Ferulic acid (phenolic acids), quercetin (flavonols), catechin (flavanols), cyanidin (anthocyanins), luteolin (flavones and other flavonoids), resveratrol (stilbenes), and oleuropein (other remaining phenolics) were used as representatives of their respective classes. A linear fitting (*R^2^* > 0.99) was built and used for quantification, and results were expressed as mg equivalents (Eq.)/g lyophilized extract (*n* = 3).

### 2.6. In Vitro Assays of Antioxidant Potential

For the evaluation of the in vitro antioxidant potential of the optimized samples, two complementary assays were used: TEAC or ABTS (as an indicator of radical scavenging activity) and FRAP (the ferric reducing antioxidant power).

For TEAC, an ABTS^+^ radical solution was prepared by reacting 7 mM ABTS solution with 2.45 mM potassium persulfate, and then the mixture was left in the dark at room temperature for 12–16 h. The ABTS+ radical solution was diluted until an absorbance of 0.70 ± 0.02 at 734 nm, and then 200 μL of radical solution were added to 20 μL of the sample (at a 1 mg/mL concentration). After 30 min of incubation in the dark at room temperature, the absorbances were read at 734 nm, and the results were expresses as milligrams of Trolox equivalents per g of freeze-dried powder (mg TE/g dw) [21,22].

For the FRAP assay (ferric reducing antioxidant power), the FRAP reagent was prepared by mixing acetate buffer (0.3 M, pH 3.6), 2,4,6-tris(2-pyridyl)-*S*-triazine (TPTZ) (10 mM), and 40 mM HCl and ferric chloride (20 mM) in a ratio of 10:1:1 (*v/v/v*). Then, 175 μL of radical solution was added to 25 μL of the sample (at a 1 mg/mL concentration), and the absorbance was read at 593 nm after a 30 min incubation at room temperature and in the dark, the activity being expressed as milligrams of Trolox equivalents per g of freeze-dried powder (mg TE/g extract) [21,22].

### 2.7. Enzyme Inhibitory Activity

The enzyme-inhibitory activity of the ORC and ERC triplicates was evaluated against α-glucosidase, tyrosinase, and acetylcholinesterase, using in vitro methods. For the α-glucosidase inhibition assay, a slightly modified previously described protocol was used [25]. In brief, 50 μL of diluted extract with different concentrations was mixed with 50 μL of enzyme (in phosphate buffer with a pH of 6.8) and 50 μL of the substrate (PNPG, 10 mM in phosphate buffer). The reaction mix was incubated at 37 °C for 15 min, and the absorbance was read at 400 nm. Acarbose was used as a positive control, and results were expressed in terms of IC_50_ (μg/mL).

For the tyrosinase inhibition assay, the protocol described by Babotă et al. was used: 25 μL of diluted extract with different concentrations was mixed with 40 μL of tyrosinase (with a 10 U/mL activity) and 100 μL of phosphate buffer with a pH of 6.8. After 15 min of incubation at room temperature, 40 μL of the substrate (L-DOPA, 2.5 mM in phosphate buffer) was added, and the reaction mixture was re-incubated for 10 min in the same conditions. The absorbance values were measured at 492 nm, and the results were expressed in terms of IC_50_ (μg/mL), using kojic acid as positive control [21,22].

A protocol based on Ellman’s method was used for the determination of the acetylcholinesterase inhibitory activity. In brief, 25 μL of diluted extract with different concentrations was mixed with 50 μL of 50 mM Tris-HCl buffer (with a pH of 8.0), 125 μL of 0.9 mM DTNB solution (in Tris-HCl buffer), and 25 μL of enzyme aqueous solution (with a 0.078 U/mL activity). The reaction mix was incubated in a dark place at room temperature for 15 min, then 25 μL of 4.5 mM ATCI solution were added and then re-incubated for 10 min. The absorbance was read at 405 nm. Galantamine was used as a positive control, and results were expressed in terms of IC_50_ (μg/mL) [22,25].

For all the enzyme inhibition assays, the determined IC_50_ values were expressed considering the dilution in the 96 wells for each sample and not as the original concentration of the re-solubilized sample.

### 2.8. Statistics and Correlation Analysis

All tested assays were made in triplicate, and the results were expressed as mean ± standard deviation. Correlogram, Pearson’s correlation coefficients, and *p*-value matrix (*p* < 0.05), evaluated for different phenolic classes and biological activities, were performed using R-studio software (version 4.1.3). Statistical analysis related to the experimental design was accomplished directly in MODDE by inspecting the replicate plot and by regression analysis [20].

## 3. Results and Discussion

### 3.1. Design of Experiments and Experimental Model Fitting

In this work, to evaluate the selection of generated D-optimal design, several criteria can be used, and in the present case two statistical parameters have been chosen as indicators: the *condition number* and the *G-efficiency*. The condition number of a design reveals its symmetry and sphericity, being expressed as a ratio between the largest and smallest values of the variability matrix. The value of this parameter shows the performance of a design prior to experimental execution, and in an ideal case it will be close to 1 (indicating orthogonality). Unfortunately, generated designs are imperfect, and the condition number should vary up to a maximum value of 8 for an efficient optimization process. On the other hand, G-efficiency parameter (Geff, expressed as percentage) shows the performance of a design in comparison to a fractional factorial design and should be above 60–70% [20]. In the present design, the condition number had a value of 4.975, and the Geff was 65.90%, indicating a high-quality and reliable D-optimal design.

**Figure 2:**
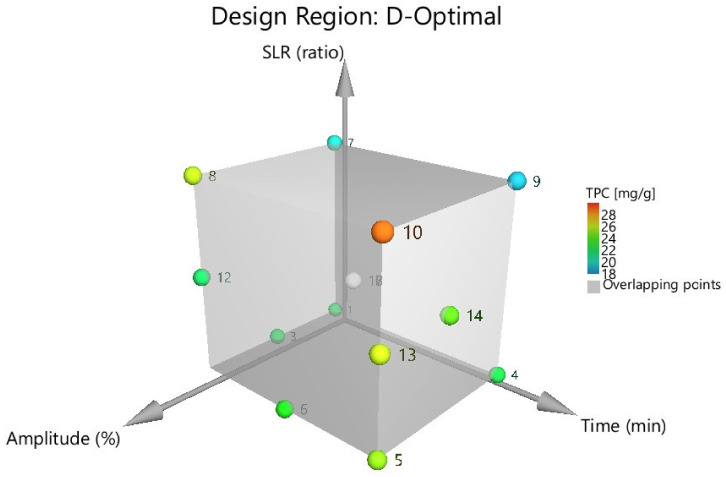
Graphical transposition of the DoE matrix (D-optimal). Each number corresponds to an experimental ID setup, as presented in Table 1.

The generated D-optimal design used for the current optimization process included three quantitative factors: ultrasound amplitude (20, 30, and 40%), exposure time (10, 30, and 50 min), and the material-solvent ratio or SLR (1:10, 1:15 and 1:20). It consisted of 15 experimental runs, from which three replicates (corresponding to the center point of the design) were performed for the estimation of the process reproducibility. Experimental runs were randomized to reduce the risk of systematic errors. As for response, TPC has been quantified for every experimental run (Table 1). The graphical transposition of the DoE matrix is presented in Figure 2.

After all experimental runs have been implemented, the determined responses were centralized and introduced in the design worksheet, allowing the analysis of the experimental data through multiple linear regression (MLR). Two relevant statistical parameters were evaluated in the first place, namely, the *R^2^* (indicating the goodness of fit of the model) and the *Q^2^* (indicating the goodness of prediction or the predictive power of the model) [20]. Furthermore, the reproducibility of the model has been assessed (based on the values of the three replicates), along with the relative standard deviation (RSD) and the model validity. The value for each parameter can be observed in Table 2.

For an extremely good model, the values of *R^2^*and *Q^2^* should be as close to 1 as possible, and for this model to be valid the difference between these two parameters cannot be more than 0.2 to 0.3. At the same time, a reproducibility higher than 0.5 should be detected for a valid model [20]. In the present design, the values of 0.986 for *R^2^*, 0.917 for *Q^2^* (with a difference of 0.069), and 0.951 for reproducibility indicate a statistically good and valid model. Moreover, a similar statistical profile has been observed in previous successful optimization studies [22,26].

### 3.2. Effects of Process Variables on the Extracted TPC

For the studied variables, the regression coefficients have been automatically established, and the following quadratic equation has been obtained (Equation (1)):

(1) $$Y=23.42+2.07X_{1}+0.95X_{2}+0.49X_{3}+1.07X_{1}X_{2}+1.74X_{1}X_{3}-0.41X_{2}X_{3}-1.42X_{1}^{2}+0.88X_{3}^{2}$$

where Y is the dependent variable (TPC); 23.42 is the model constant; 2.07, 0.95, 0.49 represent linear coefficients; 1.07, 1.74, −0.41 are interaction coefficients between two factors; and −1.42 and 0.88 are quadratic coefficients. X_1_, X_2_, and X_3_ represent the multilevel factors that have been used, extraction amplitude (%), exposure time (min), and sample-to-liquid ratio (SLR), respectively.

**Figure 3:**
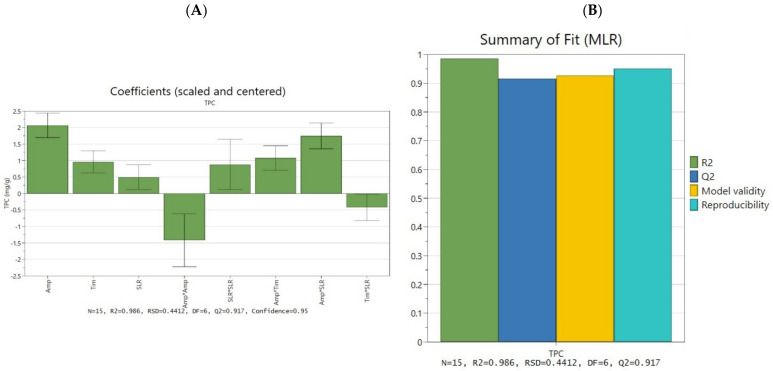
Regression analysis and model interpretation for UAE optimization process; (**A**) scaled and centered coefficient plot of the process parameter influence; (**B**) the summary of fit plot for the optimization model.

The obtained equation coefficients support the understanding of the influence of each experimental factor on the quantified response (TPC). This influence has been plotted using scaled and centered coefficients, as presented in Figure 3A, and the summary of fit is shown in Figure 3B. As the coefficient plot suggests, each extraction parameter had an influence on the TPC, but there was a difference in the magnitude of this influence. Moreover, since the chosen model was a quadratic one, the plot allowed the study of the interaction of factors (represented as a product between main factors) and of the quadratic terms.

**Figure 4:**
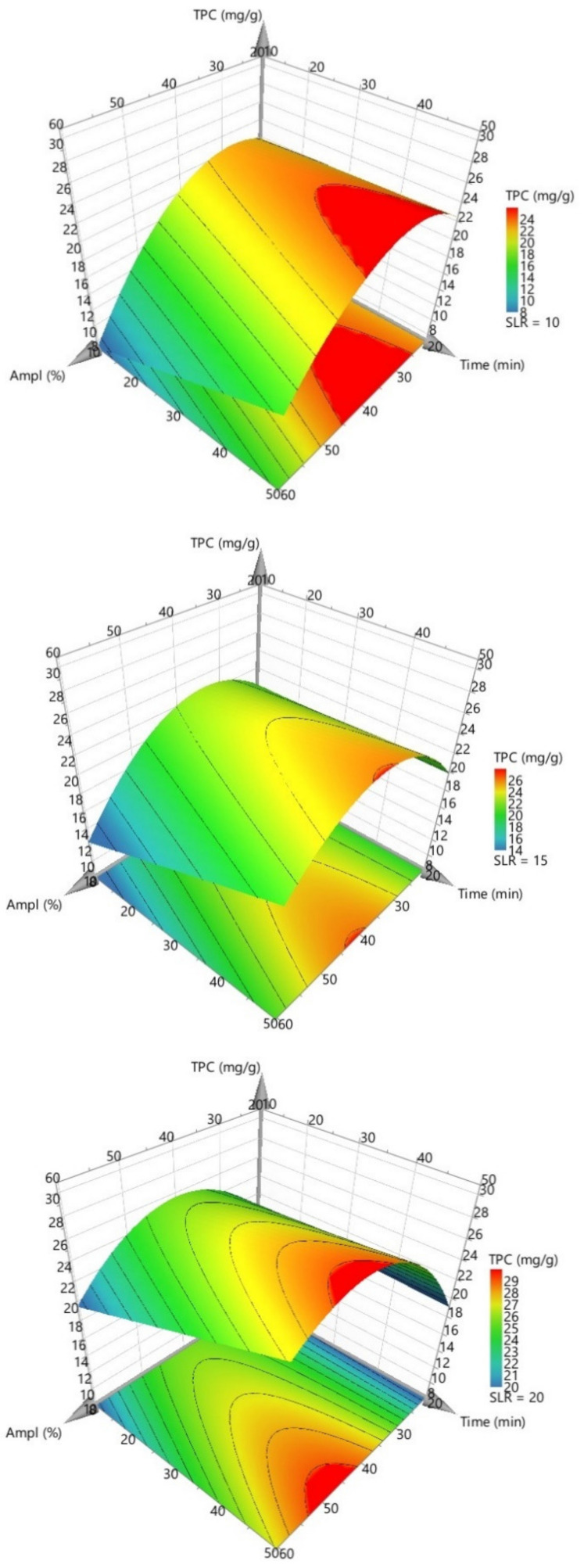
Response surface plots for TPC (mg/g) in three cases of SLR (ratio): 1:10, 1:15, and 1:20.

The highest observed influence is related to the ultrasound amplitude, and we have identified an important interaction between amplitude and time, respectively, between amplitude and SLR. As noticeable in the three-dimensional response surface plots presented in Figure 4, the best TPC results are obtained when an intermediate ultrasound amplitude is used (in the range 40–50% of amplitude, corresponding to 11.5–15.5 W of ultrasonic power), and the highest yield was identified for a 1:20 SLR. This result is supported by previous studies, high-power ultrasounds usually exhibiting a negative effect on the polyphenol release, probably due to a significant change in the chemical composition [27,28].

Accordingly, our attention was further focused on determining the significant factor interactions identified for the present model, namely, between ultrasounds amplitude and the other factors, exposure time and SLR, respectively. From our observations, these interactions suggest that there is a synergistic effect between amplitude and the two aforementioned factors. As the response surface plots in Figure 4 suggest, the TPC values are higher when both working parameters are increased simultaneously. The fact that extraction yield is improved when less material (in comparison to the solvent) is present can be attributed to a decreased density of the extraction medium, which can promote the propagation of ultrasound waves and reduce the attenuation effect [27]. This trend has also been identified for our extracts, where a higher solvent-to-material ratio (1:20), with a reduced density, showed a more efficient polyphenol extractive yield. Finally, two significant quadratic interactions have been identified which show that the ultrasonic amplitude and SLR influence was not linear. In the present study, the quadratic interaction for exposure time was insignificant.

#### Process Optimization

Using the MODDE optimizer function, which is able to find an experimental setpoint functioning as the best possible solution to the process equation [19], an objective set to maximize has been set. To maximize the extraction’s yield as much as possible, we have allowed a higher limit for the amplitude, because the 3.2 mm tapered microtip can induce a maximum of 70% ultrasound amplitude, since this was the most significant extraction factor. Further, we have introduced a range of desired TPC values (minimum 24 mg/g, maximum 34 mg/g, and a target value of 30 mg/g), taking into account the fact that the experimental determinations varied from 19.05 to 28.8 mg/g (Table 1). The predicted and experimentally measured values of TPC for the optimized samples, along with ERC samples results, are presented in Table 3.

Even though UAE can act as an efficient technique for obtaining plant extracts enriched with bioactive phytochemicals, it can be used as a good method only when an adequate combination of parameters is applied. Furthermore, it has been observed that there is no proportionality between rising the values of extraction parameters and the extraction yields, and usually there is a dependent increase of response, followed by a decrease or a steady state [10,27]. For example, in the case of UAE and temperature extraction of polyphenols, temperatures above 50 °C can induce degradation processes, resulting in an inadequate phenolic content [27]. Subsequently, the results of the experimental design allowed the establishment of possible optimal values for our work parameters, as following: 50% ultrasound amplitude (corresponding to an average power of 15.5 W), 50 min of exposure, and 1:20 SLR (with a predicted TPC value of 29.54 mg/g for the optimal extract). By applying the same process, but with the optimal parameters of extraction, we confirmed the predicted data for ORC, obtaining three TPC values for the final extracts: 29.74 ± 0.64 mg/g (O1), 30.73 ± 0.75 (O2), and 29.33 ± 0.71 mg/g (O3), respectively, with an average of 29.37 mg/g. The optimization process showed an overall recovery of 99.42%.

Furthermore, after applying the same process in association with enzymatic pre-treatment, we have obtained slightly higher TPC values for ERC: 32.08 ± 0.15 mg/g (E1), 33.86 ± 0.56 (E2), and 31.97 ± 1.71 mg/g (E3), respectively, with an average of 32.64 mg/g, showing a difference of 3.27 mg/g in TPC values between ERC and ORC. As previously stated, the activity of the enzymatic blend has been lately used as a method of increasing the yield of extracted polyphenols, alone or mixed with other modern techniques (including UAE). The higher values obtained for ERC show that a higher quantity of polyphenols have been released in the medium, probably due to the enzyme’s ability to liberate cell wall-bound polyphenols, caused by a hydrolytic attack on pectin, cellulose, and hemicellulose [17,29].

### 3.3. Total Phenolic Content (TPC)

As previously stated, TPC has been used as a response in the optimization process but also as a method for the characterization of the optimized extracts (ORC and ERC), the latter being represented in Table 3. This parameter has been chosen for the optimization study because it is frequently correlated with the antioxidant power of samples. Moreover, RC is known for possessing high quantities of polyphenolic compounds [30]. Kılıçgün et al. noticed that for RC pseudo-fruit infusions with different concentrations, there is a correlation between the TPC and the reducing power and H_2_O_2_ and $O_{2}^{-}$ scavenging activity [9]. Moreover, Daels et al. have concluded that RC exhibits in vivo and ex vivo inhibitory effects against H_2_O_2_ and superoxide anion in a dose-dependent manner [31]. Regarding the UAE of polyphenols from RC, a previous optimization study developed by Ilbay et al. concluded that a maximum of 47.23 mg GAE/g could be obtained with a combination of 40% ethanol, 50 °C, and 81.23 minutes of exposure time, employing an ultrasonic bath (40 kHz). To the best of our knowledge, this is the first study aiming to determine the optimal experimental condition for obtaining the highest TPC value for UAE water extraction in the case of rosehip by means of varying ultrasound parameters. Moreover, this is the first study describing the effect of EAE pre-treatment on the phytochemical profile of RC pseudo-fruits. To further improve the understanding of the influence of extraction procedure on the chemical composition of RC extracts, we focused our attention on flavonoids and other classes of phenolics, such as phenolic acids, anthocyanins, and stilbenes.

### 3.4. Untargeted Phenolic Profiling of ERC and ORC Extracts

In this work, the UHPLC-HRMS phenolic profiling allowed us to putatively annotate several compounds, including 50 anthocyanins, 81 flavones and derivatives, 24 flavan-3-ols, 51 flavonols, 24 lignans, 67 tyrosol derivatives, 45 phenolic acids, and 11 stilbenes. The compounds annotated are reported in Supplementary Materials together with their relative abundance values, isotopic MS, and MS/MS spectra. Overall, flavonoids were found as the most abundant class of phenolics (206 compounds), followed by lower molecular weight phenolic compounds and phenolic acids (including both hydroxycinnamics and hydroxybenzoics). Additionally, 82 phenolics were structurally confirmed according to MS/MS spectra reported in the comprehensive Food Database. Among the most abundant compounds for each class, we found cyanidin 3-O-(6″-succinyl-glucoside) (anthocyanins), 5-hydroxy-3,3′,7,8-tetramethoxy-4′,5′-methylenedioxyflavone (flavones), epigallocatechin 3-*p*-coumarate (flavan-3-ols), morin (flavonols), trachelogenin (lignans), 8-methoxy-6,7-methylenedioxycoumarin (other phenolics), gallic acid (phenolic acids), and 3′-hydroxy-3,4,5,4′-tetramethoxystilbene (stilbenes) (Supplementary Materials). As the next step, the annotated phenolics were quantified according to pure standard compounds representing the phenolic classes considered.

**Figure 5:**
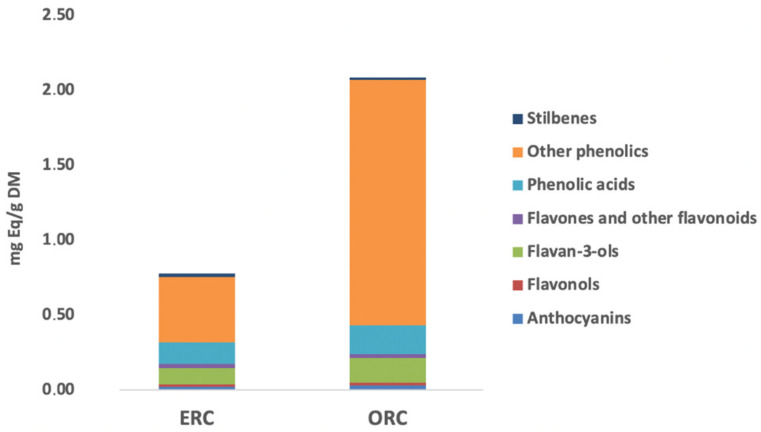
Total cumulative phenolic content of both ERC and ORC sample extracts. The results are expressed as mg phenolic equivalents (Eq.)/g dry matter (DM).

**Figure 6:**
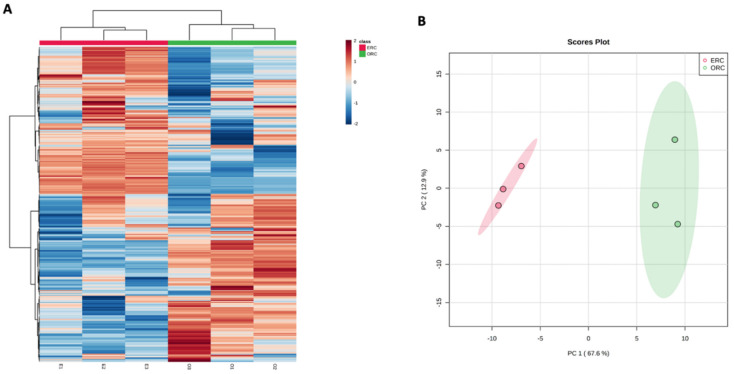
Unsupervised multivariate statistics built considering the phenolic profiles of ERC and ORC sample extracts. (**A**) = heat map based on not averaged hierarchical cluster analysis; (**B**) = principal component analysis score plot.

As it can be observed in Figure 5, it was evident that ORC was characterized by a higher cumulative phenolic content when compared with ERC, being 2.09 vs. 0.78 mg/g, respectively. Interestingly, we found that the UAE allowed us to recover about a four-fold higher content of lower molecular weight phenolics (i.e., 1.64 mg/g) when compared with the combination of EAE and UAE (i.e., 0.44 mg/g). Regarding the other classes, no significant differences were recorded by looking at the semi-quantitative values (Figure 5). The differences between the two extraction methods were then inspected by using an unsupervised multivariate statistical approach based on both hierarchical clustering (HCA) and principal component analyses (PCA) (Figure 6A,B).

The HCA heat map, built considering the log2 fold-change variation of each phenolic compound across each sample replicate, allowed us to clearly discriminate the ERC vs. ORC samples, highlighting some cluster of phenolics particularly up- and/or down-accumulated in both samples, thus confirming a clear impact of the extraction step on the phytochemical profile of *R. canina* pseudo-fruits (Figure 6A). Besides, the ability of UAE and combined EAE + UAE to affect the phenolic profile of *R. canina* pseudo-fruits was evaluated by using a PCA approach. As can be observed from the score plot represented in Figure 6B, the two principal components (PC1 and PC2) were able to cumulatively explain 80.5% of the total variability, thus confirming the ability of phenolics to be potential markers of the extraction processes under investigation.

Finally, a volcano plot analysis (combining one-way ANOVA and fold-change analyses) was used to unravel the exclusive phenolic markers of both extraction methods. Overall, as reported in Supplementary Materials, among the compounds characterized by the highest up-accumulation values in ERC vs. ORC, we found 3,4,5,4′-tetramethoxystilbene (Log2FC = 9.01), followed by 5-pentacosenylresorcinol (Log2FC = 8.91), deoxyschisandrin (Log2FC = 8.69), and 5-tricosylresorcinol (Log2FC = 8.49). In addition, a total of 45 compounds were recorded as the markers of the combined EAE + UAE system, with a great abundance of flavonoids (27 compounds), followed by other phenolics, phenolic acids, and lignans. Regarding those samples extracted by UAE, a total of 44 phenolic compounds were recorded (with a great abundance of flavonoids, i.e., 31 compounds), with chrysoeriol 7-O-(6″-malonyl-glucoside) (belonging to flavones) exhibiting the highest variation when comparing ORC and ERC samples (Log2FC value = 7.37). Regarding other phenolics class, ORC promoted the highest and significant recovery of five compounds, namely, acetyl eugenol, lithospermic acid, 3,4-DHPEA-EA, and two alkylresorcinols (i.e., 5-nonadecenylresorcinol and 5-heneicosylresorcinol) (Supplementary Materials).

Looking at some works available in literature on *R. canina L.* (dog rose) fruits, Polumackanycz et al. identified only 12 phenolic compounds (mainly phenolic acids) by using LC-DAD/ESI/MS, namely gallic acid, protocatechuic acid, vanillic acid, chlorogenic acid, syringic acid, *p*-coumaric acid, ferulic acid, sinapic acid, rutin, rosmarinic acid, cinnamic acid, and quercetin [32]. Similarly, Liaudanskas et al. identified only 10 compounds in *Rosa* L. fruit samples, such as caffeic acid, epicatechin, catechin, quercetin, chlorogenic acid, phloridzin, epicatechin-gallate, kaempferol-3-glucoside, quercitrin, and rutin [8]. Therefore, in our comprehensive investigation (based on a high-resolution and detailed untargeted phenolic profiling), we provided new insights into the phytochemical composition of *R. canina L*. pseudo-fruits, also showing a higher ability of UAE to promote the extraction of lower molecular weight phenolic compounds, when compared with a combined EAE + UAE treatment. Overall, EAE is based on the degradation or disruption of plant cell wall components, which causes the release of bound phenolic compounds and the release of polyphenols present in cell vacuoles by processes of diffusion [33]. Some previous studies attributed the efficiency of EAE to the specificity of these biomolecules for their substrate, which, in addition to the extraction of phenolic compounds, may increase the extract’s bioactivity by hydrolysis of higher molecular weight compounds to lower molecular weight compounds. Among the polysaccharides forming the cell walls of fruits and vegetables, the three most important ones are cellulose, hemicellulose, and pectin. Using specific enzymes such as cellulases, hemicellulases, xylanases, and pectinases to increase the extraction yield of phenolic compounds, it can lead to the conclusion that the results are very variable, not always causing an increased yield [34]. According to the literature, the reason behind these conflicting results is still not fully understood. One possible explanation is due to the binding between phenolic compounds and cell wall polysaccharides, according to hydrogen bonds, hydrophobic, and ionic [35]. Besides, it is also well known that polyphenols can interact with proteins, and then enzymes, according to covalent and non-covalent bonds. These interactions can affect protein stability, causing their precipitation via either multisite interactions or multidentate interactions, depending on the molar ratio of phenolic compound/protein [34]. Therefore, our findings on the untargeted phenolic profile of *R. canina* pseudo-fruits seem to suggest that the enzymatic pre-treatment was responsible for a destabilization of glycosylated phenolics followed by potential interactions with other matrix components.

### 3.5. In Vitro Antioxidant Capacity

The antioxidant capacity of the ORC and ERC samples has been assessed through two in vitro assays (ABTS or TEAC and FRAP), and the results are shown in Table 4.

**Figure 7:**
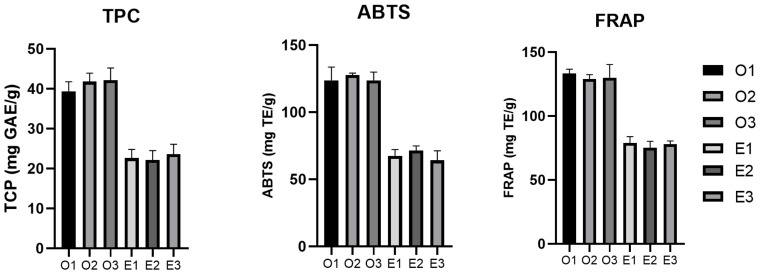
Graphs representing the antioxidant activity (through ABTS and FRAP assays) and the TPC for the obtained freeze-dried extracts. Results expressed as mean ± standard deviations of three parallel measurements.

For ORC, the antioxidant activity of the freeze-dried extracts was similar in the case of both assays (125.15 mg TE/g dw for TEAC and 130.81 mg TE/g dw for FRAP, expressed as average values), and for ERC there was an analogous trend (67.72 mg TE/g dw for TEAC and 71.53 mg TE/g dw for FRAP, expressed as average values), TEAC values being slightly lower than FRAP values. The similarity in activity for these two assays has been previously confirmed by other studies that sought to detect the antioxidant potential of RC extracts [36,37]. Comparing the two types of extraction, ORC showed a higher antioxidant activity in comparison to ERC, as shown in Figure 7.

### 3.6. Enzyme Inhibitory Activity

ORC and ERC extracts were evaluated for the inhibitory activity against α-glucosidase, tyrosinase, and acetylcholinesterase, the results being presented in Table 5. The most relevant inhibitory activity was identified for α-glucosidase (with an IC_50_ of 2.41 mg/mL for ORC and 2.47 mg/mL for ERC, expressed as average for the triplicate samples), showing an approximative 8% of inhibition activity of the positive control. On the other hand, the extracts showed a relatively low antityrosinase and anticholinesterase activity in comparison to the positive controls. Given the fact that glucosidase inhibitors are involved in the therapeutical approach of diabetes, our results regarding the inhibitory activity against α-glucosidase support the idea that rosehip could be used for its antidiabetic potential. This theory is supported by previous studies, in which different types of RC extracts have shown in vivo or in vitro inhibitory activity [13,38,39].

### 3.7. Pearson’s Correlations

**Figure 8:**
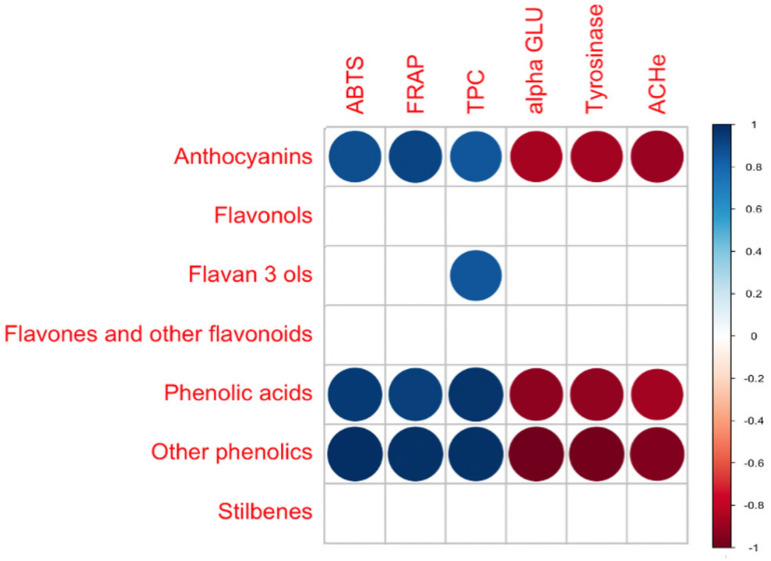
Correlogram considering the significant phenolic classes annotated (i.e., anthocyanins, flavones, flavonols, flavan-3-ols, phenolic acids, other phenolics, and stilbenes) and the measured bioactivity (i.e., ABTS and FRAP activity, followed by alpha-glucosidase, tyrosinase, and acetylcholine-esterase inhibitions).

Pearson’s correlation coefficients (r) were then inspected to evaluate those phenolic classes better correlating with the different assays (i.e., both in vitro antioxidant and enzymatic inhibition potentials). Overall, the obtained correlogram (Figure 8) revealed that anthocyanins, phenolic acids, and lower molecular weight compounds (other phenolics), established the maximum number of correlations with the different assays (i.e., six significant correlations; *p* < 0.05), whilst only one significant correlation was outlined for the class of flavan-3-ols. It was interesting to notice that, under our experimental conditions, phenolic compounds were only able to explain the in vitro antioxidant potential of the extracts (ORC and ERC), with anthocyanins, phenolic acids, and other phenolics establishing significant correlations with both ABTS and FRAP assays. On the other hand, the enzymatic inhibition showed negative and significant correlations with these phenolic classes, thus suggesting that there are other unidentified classes of bioactive compounds (different from phenolics) that were responsible for these activities. Finally, regarding the correlation coefficients between phenolic classes and the TPC (as obtained from in vitro spectrophotometric assay), phenolic acids (*p* < 0.01; r = 1) and flavan-3-ols (*p* < 0.05; r = 0.99) were the most correlated classes of compounds.

## 4. Conclusions

In this study, we have developed an optimization of ultrasound-assisted water extraction of polyphenols from the powder of *Rosa canina* L. pseudo-fruits (rosehip), in comparison to a combination of this method and enzymatic-assisted extraction as pre-treatment step. A computed D-optimal design allowed us to find that 50% amplitude, 50 min of exposure, and 1:20 ratio could serve as a good candidate for optimal working conditions. Moreover, DoE approach allowed the assessment of the interactions between the working parameters and the quality of the final extract. Our initial findings showed that EAE and UAE resulted in a higher recovery of total phenolics (32.64 mg/g in average) in comparison to using UAE alone (29.37 mg/g in average), yet the composition of extracts showed a high difference after the freeze-drying process.

An UHPLC-HRMS method was used to ascertain the phenolic profile of ORC and ERC, revealing a high content of flavonoid-type compounds, and gallic acid was the most abundant compound in both cases. In addition, the phytochemical profile was correlated with antioxidant and enzyme inhibitory activities of the two types of extracts, highlighting the importance of rosehip as a source of beneficial bioactive compounds. Finally, our results show that using exclusively ultrasound-assisted extraction resulted in a higher cumulative phenolic content and a higher antioxidant and enzyme-inhibitory activity in the case of freeze-dried extracts, in comparison to using this method in association with enzymatic extraction.

## Figures and Tables

**Table 1:** DoE matrix and TPC values (expressed as mg GAE/g of plant material) for the extracts corresponding to each experimental run.

Exp No	Exp ID	Amplitude (%)	Average Power (W)	Time (min)	S-L Ratio (1:*n*)	TPC (mg/g)
1	N1	20	3.97	10	10	21.96
2	N3	30	7.27	10	10	22.03
3	N4	20	3.97	50	10	22.27
4	N5	40	11.51	50	10	25.08
5	N6	40	11.51	30	10	23.10
6	N7	20	3.97	10	20	19.97
7	N8	40	11.51	10	20	25.69
8	N9	20	3.97	50	20	19.05
9	N10	40	11.51	50	20	28.80
10	N12	40	11.51	10	15	21.80
11	N13	40	11.51	50	15	25.94
12	N14	30	7.27	50	15	24.26
13	N16	30	7.27	30	15	24.22
14	N17	30	7.27	30	15	23.26
15	N18	30	7.27	30	15	23.32

Notes: S-L (sample-to-liquid); 1:10 corresponds to 1 g of material and 9 g of solvent (water).

**Table 2:** The values of parameters used to evaluate experimental model fitting.

Parameter	R^2^	R^2^ Adj.	Q^2^	RSD	*n*	Model Validity	Reproducibility
TPC	0.986	0.967	0.917	0.441	15	0.926	0.951

**Table 3:** Overview of predicted and experimentally measured TPC values obtained for the ORC and the experimentally measured values for ERC triplicates for the original extracts before the freeze-drying step. Results expressed as mean ± standard deviations of three parallel measurements.

TPC (mg GAE/g dw)	ORC			ERC		
	O1	O2	O3	E1	E2	E3
**Experimentally Measured**	29.74 ± 0.64	30.73 ± 0.75	29.33 ± 0.71	32.08 ± 0.15	33.86 ± 0.56	31.97 ± 1.71
**Average**	29.37			32.64		
**Predicted**	**Minimum**	**Target**	**Maximum**	**DoE Predicted**	**Probability of Failure**	**Recovery**
	24	30	34	29.54	0.21%	99.42%

**Table 4:** Overview of TPC, TFC, and in vitro antioxidant capacity values measured for the ORC and ERC triplicates. Results expressed as mean ± standard deviations of three parallel measurements.

Assay	ORC			ERC		
	O1	O2	O3	E1	E2	E3
TPC (mg GAE/g dw)	39.39 ± 2.37	41.80 ± 2.14	42.12 ± 3.09	22.66 ± 2.13	22.14 ± 2.37	23.62 ± 2.46
TEAC (mg TE/g dw)	123.84 ± 9.82	127.75 ± 1.49	123.86 ± 6.07	67.51 ± 4.70	71.51 ± 3.37	64.14 ± 7.13
FRAP (mg TE/g dw)	133.41 ± 3.25	128.95 ± 3.48	130.08 ± 10.24	78.95 ± 4.95	75.28 ± 5.06	78.05 ± 2.46

**Table 5:** Overview of the in vitro enzyme inhibitory activity values determined for the ORC and ERC triplicates.

Enzymatic Assay	ORC			ERC		
	O1	O2	O3	E1	E2	E3
α-Glucosidase (IC_50_, mg/mL)	2.45	2.42	2.36	2.53	2.35	2.55
	Acarbose: 0.1946					
Tyrosinase (IC_50_, mg/mL)	3.02	2.83	3.26	3.82	4.09	3.56
	Kojic acid: 0.01395					
Acetylcholinesterase (IC_50_, mg/mL)	1.09	4.12	1.23	6.19	7.50	6.63
	Galantamine: 2.23 × 10^−5^					

## Data Availability

Data are contained within the article or Supplementary Materials.

## Supplementary Materials

The following supporting information can be downloaded at: https://www.mdpi.com/article/10.3390/antiox11061123/s1, Table S1: LC-MS Dataset; Table S2: Volcano ERC vs. ORC; Table S3: Pearson’s correlation.
